# Zika virus syndrome, lack of environmental policies and risks of worsening by cyanobacteria proliferation in a climate change scenario

**DOI:** 10.11606/s1518-8787.2020054002159

**Published:** 2020-10-20

**Authors:** Sofia Lizarralde Oliver, Helena Ribeiro

**Affiliations:** I II Universidade de São Paulo Faculdade de Saúde Pública Departamento de Saúde Ambiental São PauloSP Brasil Universidade de São Paulo. Faculdade de Saúde Pública. Departamento de Saúde Ambiental. São Paulo, SP, Brasil

**Keywords:** Zika virus infection, Neurotoxins, Cyanobacteria, Water Pollution, Sanitation, Environmental Health

## Abstract

Almost half of the Brazilian population has no access to sewage collection and treatment. Untreated effluents discharged in waters of reservoirs for human supply favor the flowering of cyanobacteria – and these microorganisms produce toxins, such as saxitoxin, which is a very potent neurotoxin present in reservoirs in the Northeast region. A recent study confirmed that chronic ingestion of neurotoxin-infected water associated with Zika virus infection could lead to a microcephaly-like outcome in pregnant mice. Cyanobacteria benefit from hot weather and organic matter in water, a condition that has been intensified by climate change, according to our previous studies. Considering the new findings, we emphasize that zika arbovirus is widespread and worsened when associated with climate change, especially in middle- or low-income countries with low levels of sanitation coverage.

## INTRODUCTION

The outbreak of Zika syndrome, as the epidemic that occurred in Brazil in 2015 and 2016 became known worldwide, was considered a public health emergency. In October 2015, Brazilian scientists reported an association between Zika virus infection and microcephaly in newborns. Virus infection during pregnancy was related to malformation and congenital microcephaly.

Outbreaks and evidence of Zika infection transmitted by Aedes-type mosquitoes have been reported in 86 countries^1^. In Brazil, the responsible was *Aedes aegypti*, which is found in tropical and subtropical regions.

A study published in September 2019^2^ was the first to associate the outcome of congenital malformations caused by zika with the cofactor of contamination by saxitoxins in water for human supply. Saxitoxins are one of the most potent and common neurotoxic found in nature.

Although other regions of Brazil reported a higher number of zika cases, the Northeast region, located in low latitudes and with a mostly warm climate, recorded the highest percentage of brain imaging tests reporting microcephaly (88.4%)^2^; in the Southeast, only 8.7% of the same tests presented malformation.

The Northeast region goes through periods of drought that favor the proliferation of cyanobacteria in lentic water bodies, such as lakes and dams. Cyanobacteria produce neurotoxins, such as saxitoxins, with implications for human and animal health. Such neurotoxins are water soluble and permeate the common water treatment systems. The Outbreak of Zika syndrome coincided with a major drought in the region between 2012 and 2016^3^. Decrease in nebulosity, characteristic of dry seasons, concentration of nutrients from untreated effluents and lower volume of water and increase in atmospheric temperature and water column allow greater flowering of cyanobacteria. Consequently, the concentration of cyanotoxins, such as saxitoxins, increases.

### Cyanobacteria and Public Health

Cyanobacteria are well known for their potential to produce cyanotoxins, such as saxitoxins (neurotoxins), which can cause rapid death of animals by respiratory arrest. The effects on human health can vary from bowel, liver and neuromuscular disorders and allergic reactions to cancer and death; and a new threat is now added.

The article “The cyanobacterial saxitoxin exacerbates neural cell death and brain malformations induced by zika virus”^2^ associated the highest incidence of zika virus-related microcephaly cases with saxitoxin contamination of supply water reservoirs in the Northeast. The study, which evaluated the spread of cyanobacteria and saxitoxins in Brazilian regions during the Zika outbreak, confirmed the synergism between saxitoxins and Zika virus, first *in vitro* – using organoids of the human brain at concentrations of saxitoxins similar to those found in water reservoirs in the Northeast region – and subsequently accompanying pregnant female mice, which consumed water contaminated by saxitoxins and infected with zika virus during pregnancy. The authors argue that the Brazilian Northeast had a lower number of cases of Zika when compared with other regions, such as the Midwest or Southeast; however, this region had a higher incidence of microcephaly. This information led them to formulate the hypothesis that cyanobacteria in the water supply would be a causal cofactor of zika-associated microcephaly.

The second region with the highest presence of saxitoxins in water is the Southeast, but with lower values of cyanobacteria concentration. One factor to be considered is that only 26.87% of northeasterners have sewage collection services. In Southeast, this percentage increases to 78.56%.

The researchers point out that the chronic exposure of mice to saxitoxins before and during Zika virus infection resembled what could have occurred with fetuses in northeastern Brazil.

## DISCUSSION

The article “Challenges regarding water quality of eutrophic reservoirs in urban landscapes: a mapping literature review,”^5^ a literature review on the presence of cyanobacteria in urban reservoirs in the world, showed that this species is a constant problem, which may represent an even more serious threat to public health, since many reservoirs are, or have the potential to be, used in the water supply due to the proximity of populations.

The causes of cyanobacteria proliferations in urban environments are mainly the disposal of untreated domestic sewage in water reservoirs and surface runoff water from soils. Currently, only 52.36% of the Brazilian population has access to sewage collection; therefore, about 100 million Brazilians lack this service^6^.

Climate variations should also be considered, since they increase temperature and intensify the existence of organic matter in water, conditions that benefit cyanobacteria, according to our previous studies^7^. This factor may represent an expanded and more severe health risk for the population supplied by water with the presence of cyanobacteria when associated with the expansion of arboviruses, especially those transmitted by *Aedes aegypti,* such as zika virus.

## FINAL REMARKS

The same climatic conditions favors both the proliferation and dispersal of mosquitoes and the flowering of cyanobacteria, whose toxins may be correlated with congenital zika syndrome. The risk to dense urban populations can be greatly minimized if basic sanitation actions, which have been delayed for so many decades, are carried out urgently and comprehensively. Preventing water contaminated due to the lack of sanitation from entering the dams and supply reservoirs is the fastest and most effective way of benefiting the population in all aspects. Congenital Zika syndrome may be just the latest warning of the need for a broad sanitation policy for Brazil and other low- and middle-income regions in the world.
