# Supplementary material for: Zika virus syndrome, lack of environmental policies and risks of worsening by cyanobacteria proliferation in a climate change scenario
Source: Rev Saude Publica. 2020 Oct 20;54:83. doi: 10.11606/s1518-8787.2020054002159 (PMC7575220; doi:10.11606/s1518-8787.2020054002159)
Supplement: Translation

# Translation: Síndrome do zika vírus, carência de políticas ambientais e riscos de agravamento por proliferação de cianobactérias em um cenário de mudanças climáticas

**Authors:** Sofia Lizarralde Oliver, Helena Ribeiro

**Affiliations:** I II Brasil Universidade de São Paulo. Faculdade de Saúde Pública. Departamento de Saúde Ambiental. São Paulo, SP, Brasil

**Keywords:** Infecção por Zika virus, Neurotoxinas, Cianobactérias, Poluição da Água, Saneamento, Saúde Ambiental

## Abstract

No Brasil, quase metade da população não tem acesso a coleta e tratamento de esgotos. Os efluentes não tratados, devido a contaminação das águas de reservatórios para abastecimento humano, geram florações de cianobactérias – e esses microrganismos produzem toxinas, como a saxitoxina, uma neurotoxina bastante potente e presente nos reservatórios da região Nordeste. Estudo recente confirmou que a ingestão crônica de água contaminada com neurotoxinas associada a infeção pelo zika vírus em camundongos prenhes poderia levar a desfecho semelhante a microcefalia. Cianobactérias são beneficiadas por tempo quente e matéria orgânica na água, e essas condições estão sendo intensificadas pelas mudanças climáticas, segundo nossos estudos anteriores. Aqui, ressaltamos que, à luz dos novos achados, a arbovirose do zika, associada às mudanças do clima, se amplifica e se agrava, sobretudo em países de média ou baixa renda, com baixos níveis de cobertura de saneamento.

## INTRODUÇÃO

O surto de síndrome do zika, como ficou conhecida mundialmente a epidemia ocorrida no Brasil em 2015 e 2016, foi considerado uma emergência de saúde pública. Em outubro de 2015, cientistas brasileiros relataram uma associação entre infecção pelo vírus zika e microcefalia em recém-nascidos. A infecção pelo vírus durante a gestação foi relacionada a má-formação e microcefalia congênita.

Surtos e evidências de infecção por zika transmitida por mosquitos do tipo Aedes foram relatados em 86 países^1^. No Brasil, o responsável foi o *Aedes aegypti*, que ocorre em regiões tropicais e subtropicais.

Um estudo publicado em setembro de 2019^2^ associou, pela primeira vez, o desfecho das más-formações congênitas provocadas pelo zika ao cofator da contaminação por saxitoxinas na água para abastecimento humano. As saxitoxinas são um dos mais potentes e comuns neurotóxicos encontrados na natureza.

Embora outras regiões do Brasil tenham notificado número maior de casos de zika, a região Nordeste, localizada em baixas latitudes e de clima majoritariamente quente, foi a que registrou maior porcentagem (88,4%) de exames de imagem cerebral reportando microcefalia^2^, ao passo que na região Sudeste só 8,7% dos mesmos exames apresentaram a má-formação.

A região Nordeste passa por períodos de seca que favorecem a proliferação de cianobactérias nos corpos d’água lênticos, como lagos, represas e açudes. As cianobactérias são capazes de produzir neurotoxinas, como as saxitoxinas, com implicações para a saúde humana e também animal. Elas são hidrossolúveis e perpassam pelos sistemas comuns de tratamento de água. O surto de síndrome do Zika coincidiu com uma grande seca na região, entre os anos 2012 e 2016^3^. Diminuição de nebulosidade, característica de épocas de estiagem, concentração de nutrientes oriundos de efluentes não tratados e do menor volume de água e aumento da temperatura atmosférica e da coluna d’água permitem maior floração das cianobactérias. Em decorrência, há elevada concentração das toxinas produzidas por elas, como as saxitoxinas.

### Cianobactérias e Saúde Pública

Cianobactérias são bastante conhecidas por seu potencial de produzir cianotoxinas, como as saxitoxinas (neurotoxinas), que podem causar morte rápida de animais por parada respiratória. Os efeitos na saúde humana podem ir de desordens intestinais, disfunções no fígado e neuromusculares, reações alérgicas, câncer e morte⁴, sendo agora acrescida uma nova ameaça.

O artigo “*The cyanobacterial saxitoxin exacerbates neural cell death and brain malformations induced by zika virus*”^2^ associou a mais alta incidência de casos de microcefalia relacionados ao zika vírus com a contaminação por saxitoxinas de reservatórios de água de abastecimento do Nordeste. O estudo, que avaliou a disseminação de cianobactérias e saxitoxinas nas regiões brasileiras durante o surto de zika, confirmou o sinergismo entre saxitoxinas e zika vírus, primeiramente *in vitro* – usando organoides do cérebro humano em concentrações de saxitoxinas semelhantes às encontradas em reservatórios de água da região Nordeste – e, posteriormente, acompanhando camundongos fêmeas grávidas, que consumiam água contaminada por saxitoxinas e infectadas pelo vírus zika durante a gestação. Os autores discutem que o Nordeste brasileiro teve menor número de casos de zika, quando comparado a outras regiões, como a Centro-Oeste ou a Sudeste, mas que apresentou maior incidência de microcefalia. Foi isso que os levou à formulação da hipótese de que as cianobactérias na água de abastecimento seriam um cofator causal da microcefalia associada ao zika.

A segunda região com maior presença de saxitoxinas é a Sudeste, mas com valores inferiores de concentração de cianobactérias. Um fator a ser considerado é que somente 26,87% dos nordestinos têm atendimento de coleta de esgotos, enquanto 78,56% da população na região Sudeste têm esgoto coletado.

Os pesquisadores destacam que a exposição crônica de camundongos a saxitoxinas, antes e durante a infecção pelo zika vírus, assemelhava-se ao que poderia ter ocorrido com os fetos no Nordeste brasileiro.

## DISCUSSÃO

O manuscrito “*Challenges regarding water quality of eutrophic reservoirs in urban landscapes: a mapping literature review*”⁵, uma revisão de literatura sobre a presença de cianobactérias em reservatórios urbanos no mundo, mostrou que esta espécie é um problema constante, que pode representar no futuro uma ameaça ainda mais séria à saúde pública, já que muitos reservatórios são, ou têm potencial para ser, utilizados no abastecimento de água, devido à proximidade de populações.

As causas das proliferações de cianobactérias em ambientes urbanos são sobretudo esgotos domésticos não tratados e água de escoamento superficial de solos. Na atualidade, somente 52,36% da população brasileira tem acesso a coleta de esgoto; portanto, cerca de 100 milhões de brasileiros não recebem esse serviço^6^.

Deve-se levar em conta ainda as variações climáticas, que aumentam a temperatura e intensificam a existência de matéria orgânica na água, condições que beneficiam as cianobactérias, conforme nossos estudos anteriores^7^. Associado à expansão das arboviroses, sobretudo as transmitidas pelo *Aedes aegypti,* como o vírus zika, esse fator pode representar um risco de saúde ampliado e mais grave para a população abastecida por água com presença de cianobactérias.

## CONSIDERAÇÕES FINAIS

As mesmas condições climáticas que favorecem a proliferação e a dispersão dos mosquitos são as que favorecem as florações de cianobactérias, com as toxinas que podem estar correlacionadas à síndrome congênita do zika. O risco para as densas populações urbanas pode ser bastante minimizado se as ações de saneamento básico, há tantas décadas proteladas, forem efetuadas com urgência e de forma abrangente. Evitar que água contaminada por falta de saneamento entre nas represas e açudes de abastecimento é a forma mais rápida e efetiva de benefício à população em todos os aspectos. A síndrome congênita do zika pode ser só o mais recente alerta para a necessidade de uma política ampla de saneamento para o Brasil e outras regiões de baixa e média renda no mundo.
